# Long-term effect of ovary removal on the joints of aged mice

**DOI:** 10.1186/s42826-026-00271-z

**Published:** 2026-04-13

**Authors:** Sofia Wüstenhagen, Lindsay Zentveld, Francesco Longo, Loise Råberg, Alexandra Stubelius, Riddhi Vyas, Anders Nguyen, Julia M. Scheffler, Mattias N. D. Svensson, Carmen Corciulo

**Affiliations:** 1 Department of Rheumatology and Inflammation, Institute of Medicine, University of Gothenburg, Gothenburg, Sweden; 2 Department of Pharmacology, Institute of Neuroscience and Physiology, University of Gothenburg, Gothenburg, Sweden; 3 Division of Chemical Biology, Department of Life Sciences, Chalmers University of Technology, Gothenburg, Sweden

**Keywords:** Aging, Hormones, Joint, Ovariectomy, Pain

## Abstract

**Background:**

Age-related diseases are medical conditions whose incidence or severity increases with advancing age. Among them, musculoskeletal diseases, like osteoarthritis, are associated with postmenopausal hormonal shifts. Ovariectomy (OVX) is a widely used animal model to mimic the decline of ovarian hormones after menopause. Most preclinical studies rely on ovariectomized young animals, questioning their relevance to faithfully replicate the complexities of the human conditions. This study examines the long-term effects of OVX on motor performance, nociceptive response, and joint morphology in adult mice, all of which are osteoarthritis-related characteristics. Seven-month-old female mice underwent OVX or sham surgery and were monitored longitudinally until 18 months of age. Motor ability was assessed using rotarod and open field tests, pain sensitivity was evaluated via Von Frey testing, and bone integrity was analyzed through micro-computed tomography (µCT). Histological evaluation of articular cartilage was performed using Safranin-O staining.

**Results:**

A transient increase in mechanical pain sensitivity was observed between 10 and 13 months post-OVX. Despite this change, OVX did not exacerbate the age-related decline in motor function. µCT revealed reduced bone mineral density in the subchondral cortical and vertebral trabecular bone in OVX mice, without significant changes in trabecular volume. Articular cartilage degeneration was similar in both experimental groups.

**Conclusions:**

These findings suggest that hormonal depletion alone may not be sufficient to drive the full osteoarthritic phenotype in aging mice. Importantly, longitudinal studies allow the capture of subtle differences in the aging process.

## Background

Animal models are widely used to replicate pathological conditions to investigate disease mechanisms or evaluate pharmacological interventions. Among experimental animals, rodents have been employed in 99% of the preclinical studies [1]. Despite physiological differences from humans and inherent limitations, rodents remain a valuable tool in biomedical research. However, the complexity of certain disorders often poses challenges for disease modeling, which can be further amplified by suboptimal experimental design, particularly when critical variables such as age, sex, and weight are not adequately considered [2]. These factors can strongly influence disease onset and progression as well as the therapeutic response.

As the global population ages [3], there is increasing emphasis on studying diseases that become more prevalent and severe with advancing age and menopause onset [4]. In women, menopause and the associated decline in ovarian hormones further elevate the risk of musculoskeletal diseases [5]. Ovariectomy (OVX), the surgical removal of the ovaries, is a commonly used method to induce a rapid decline in gonadal hormones in rodents, thereby modeling conditions associated with the postmenopausal state, such as osteoarthritis [6, 7]. Although osteoarthritis predominantly affects older adults, many preclinical studies use young animals in OVX models, failing to account for the aging context in which these diseases typically develop. While mice do not undergo menopause as human do, they do exhibit a gradual decline in fertility and mild hormonal changes beginning after 6 months of age [8]. This is in contrast to the abrupt hormonal shifts seen in women, where levels of estradiol and progesterone fluctuate during perimenopause and then stabilize at low levels post-menopause [9]. Studies using young mice might overlook age-related cellular and tissue changes that are critical for understanding disease mechanisms in the aging population.

In rodents, as in humans, aging impacts pain sensitivity [10], motor function [11], and the structure of bones [12] and cartilage [13], all key aspects of musculoskeletal diseases that the decline in gonadal hormones can further exacerbate. While substantial data exists on the effects of ovary removal in young mice, there is limited information on the long-term consequences of OVX in aged mice. Therefore, this study aims to investigate the combined effects of ovariectomy and aging on joint health in adult female mice.

## Methods

### Experimental animals

**Fig. 1:**
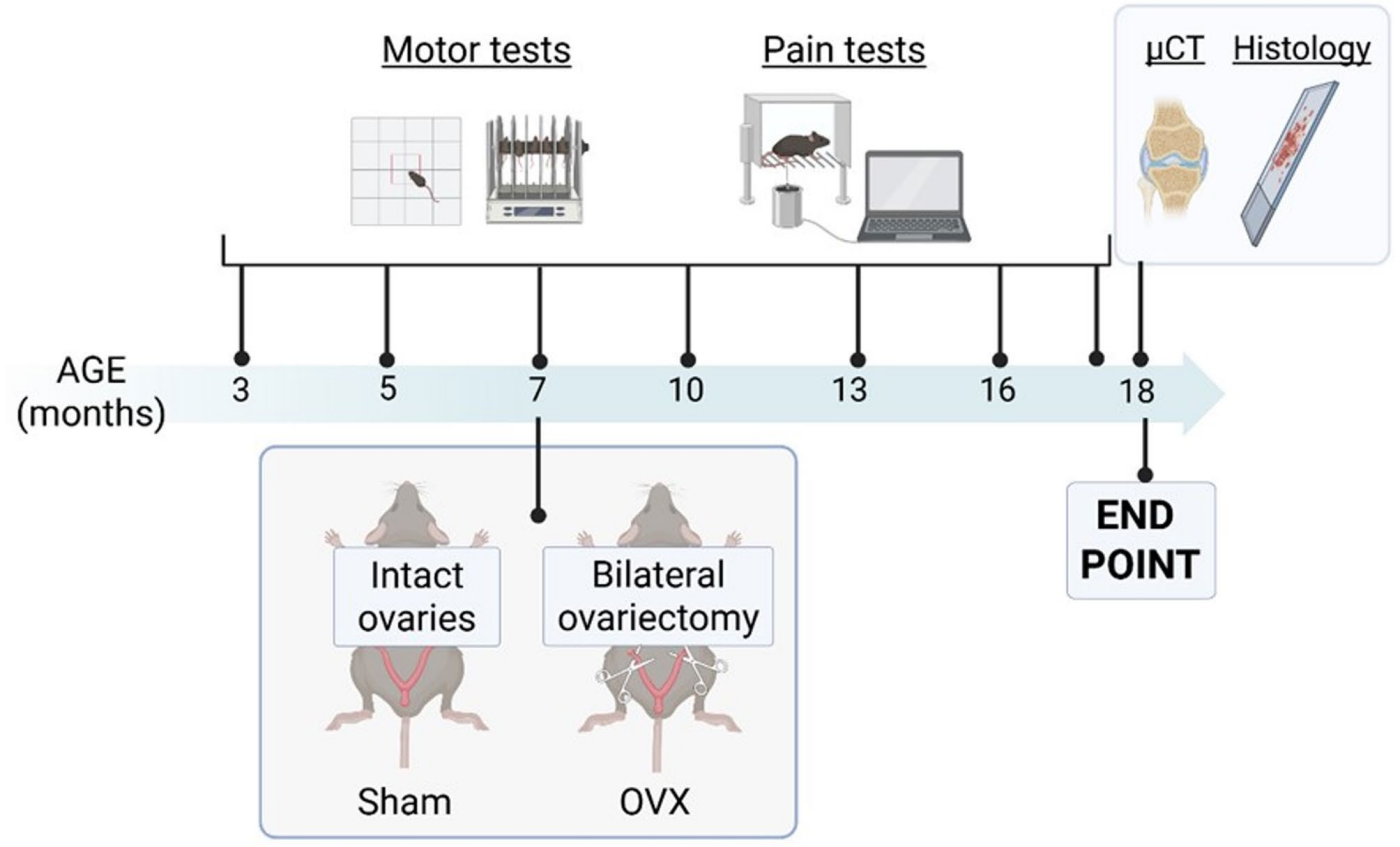
Graphical representation of the experimental setting. Ovariectomy was performed on mice at 7 months of age. Motor function and pain sensitivity were assessed longitudinally from 3 months of age until the study endpoint. Knee joints were collected post-mortem for micro-computed tomography (micro-CT) and histological analysis

Female C57BL/6J mice (Taconic, Borup, Denmark) were housed in the animal facility at the University of Gothenburg (Sweden) under standard conditions, including a 12-hour light/12-hour dark cycle. Animals were provided with soy-free laboratory chow and tap water ad libitum. Before testing, mice were acclimatized for 7 days. Experimental procedures followed the timeline outlined in Fig. 1. All procedures were conducted in accordance with ethical permit 2814 − 2020, approved by the Regional Ethical Review Board in Gothenburg, Sweden.

### Assessment of motor ability

Motor performance was evaluated using the Rotarod test (assessing forced locomotor activity) and the Open Field test (assessing spontaneous locomotor activity). Mice were acclimated in the behavioral testing room for 1 h before the start of the assessments. A rest period of 30 min was provided between trials and tests.

In the Open Field test, each mouse was placed in a 60 × 60 × 60 cm arena and allowed to explore freely. Locomotor activity was recorded for 15 min and analyzed using automated tracking software (Viewer, Sankt Augustin, Germany).

For the Rotarod test, mice were placed on a rotating rod apparatus (Panlab, Harvard Apparatus, Cornella, Spain) with a continuously accelerating speed from 4 to 40 rpm over a 5-minute period. The latency to fall was recorded for each mouse. Each group underwent three trials per day for two consecutive days.

### Assessment of the pain sensitivity

Mechanical pain sensitivity was assessed using the Von Frey filament limb withdrawal test on both hind paws. Mice were placed individually in transparent plastic boxes positioned on a metal mesh platform, allowing access to the paws. Following a 30-minute acclimation period, mechanical stimuli were applied to the mid-plantar surface of each hind paw using a Dynamic Plantar Aesthesiometer (Ugo Basile, Gemonio, Italy). The device automatically recorded the force and latency at which a withdrawal response occurred.

### Ovariectomy

Seven-month-old female mice underwent either bilateral OVX or sham surgery. Anesthesia was induced and maintained with a mixture of isoflurane (1.5–2.5%) and oxygen. The lower back was shaved and disinfected with chlorhexidine. A 1 cm midline incision was made in the skin, followed by a 5 mm incision in the peritoneum on one side to expose the ovarian fat pad. The ovary was excised via cauterization, and the peritoneal incision was closed using absorbable sutures. The procedure was then repeated on the contralateral side. The skin incision was closed with surgical clips.

In sham-operated mice, the ovarian fat pads were exposed but the ovaries were left intact. All animals received meloxicam (5 mg/mL; Boehringer Ingelheim, Ingelheim am Rhein, Germany) as postoperative analgesia. After OVX, mice from the two experimental groups were housed in separate cages to prevent hormone exposure through feces.

### Termination of the experiment and tissue collection

At the endpoint, mice were anesthetized with a combination of ketamine (Salfarm, Kolding, Denmark) and dexmedetomidine hydrochloride (Zoetis, Parsippany, NJ - USA). Once fully anesthetized, animals were euthanized by exsanguination followed by cervical dislocation. The uterus, thymus, perigonadal fat, and tibialis anterior muscle were dissected and weighed. The dry weight of the tibialis anterior was determined after overnight incubation at 60 °C. Whole knee joints were harvested for histological evaluation of cartilage integrity, and both the joints and lumbar vertebrae were subjected to micro-computed tomography (µCT) analysis.

### uCT

After sacrifice, the right hind leg was excised, and the soft tissue was carefully removed from the bone. Samples were fixed in 4% paraformaldehyde for 3 days and then stored in 70% ethanol. The subchondral area for the trabecular bone evaluation starts below the cortical bone in the epiphyseal region and extends for a longitudinal distance of 495 μm in the distal direction. The region of interest (ROI) for the measurement of the subchondral cortical bone was defined as the 742 μm area extending in the distal direction starting from the tibial plateau. The ROI for the lumbar vertebrae L3 was defined as the 641 μm area starting at 1.296 mm in the caudal direction. The selected area was evaluated in a scanning tube providing a voxel size of 4.49 μm isotropically and scanned at 50 kV, 200 mA (Skyscan 1172 scanner; Bruker MicroCT, Kontich, Belgium). The samples were kept on paper soaked in PBS to avoid dehydration. Data reconstruction and analysis of the bone features by µCT were performed using the software CtAN (1.13.2.1), Nrecon (1.6.9.8), and DataViewer (1.5.0.9, Bruker MicroCT; Kontich, Belgium).

### Histology

After the µCT analysis, the knees were decalcified in 10% EDTA (Merck KGaA, Darmstadt, Germany) for 21 days at 4 °C. Specimens were embedded in paraffin blocks, and 5-µm coronal sections of the knees were obtained. The sections were used for Safranin-O/fast green staining.

Pictures of the slides were acquired by using the microscope Nikon Eclipse 90i and the software NIS-Elements ver. 4.40 (Nikon, Melville, NY, U.S.A.). Assessment of cartilage damage was performed by evaluation of Safranin-O-stained slides. The SMASH score was determined as previously described [14]. Briefly, proteoglycan loss was assessed using a semiquantitative scoring system ranging from 0 to 3, where 0 indicates no loss (healthy cartilage) and 3 represents severe loss, characterized by complete destaining of the superficial hyaline cartilage. Three slides from both the tibia and femur were evaluated, and their scores were summed.

### Data analysis

Statistical analysis was performed using GraphPad Prism 9 software (Version 10.2.2; GraphPad Software, Inc; La Jolla, CA, USA). Results were presented as mean ± SEM. *p* < 0.05 was considered statistically significant. Statistical comparisons between two groups were conducted using an unpaired *t-test*. Data with two independent variables were analyzed using two-way ANOVA followed by Dunnett’s post hoc test. A total of 15 animals per group were included in the study. Of these, one animal from the sham group and two animals from the OVX group were sacrificed at 15 months of age due to ethical considerations related to poor health status.

## Results

### Body weight and perigonadal fat did not change in OVX mice

**Fig. 2:**
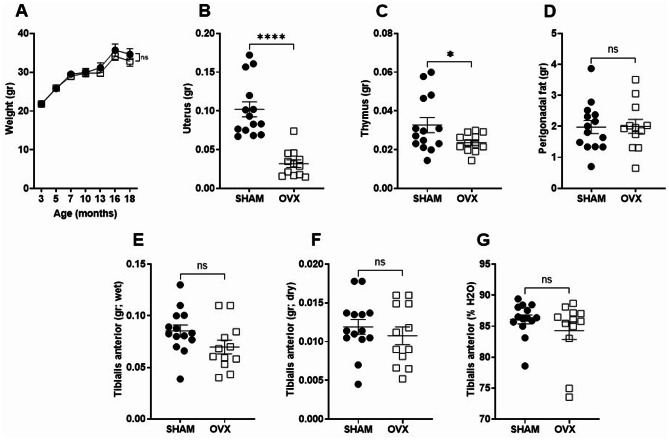
Body weight and perigonadal fat were not altered in OVX mice compared to sham-operated controls. (**A**) Body weight was monitored throughout the experiment in sham-operated and OVX mice. (**B**-**D**) Uterus, thymus, and perigonadal fat weights were measured in 18-month-old mice at the time of sacrifice. (**E**-**G**) Dry and wet weight and percentage of water content were measured in the isolated skeletal muscle tibialis anterior. Data are presented as mean ± SEM. Statistical analysis was performed using two-way ANOVA for body weight and unpaired Student’s t-test for the weight of the uterus, thymus, spleen, skeletal muscle, and perigonadal fat. *, represent statistical differences between Sham and OVX mice; *, *p* < 0.05; ****, *p* < 0.001

Body weight was monitored throughout the entire duration of the experiment. The body weight increased over time for both experimental groups. No significant differences in body weight were observed between experimental groups following OVX (Fig. 2A). Thymus, perigonadal fat, and uterine weights were recorded, as these tissues are sensitive to gonadal hormone fluctuations in young mice. While the weight of the perigonadal fat and skeletal muscle (total and dry weight) did not differ between groups (Fig. 2D, E, F), uterine and thymus weights were significantly reduced in OVX mice compared to sham-operated controls (Fig. 2B, C).

### Motor abilities decline with age but are not worsened in OVX compared to the sham-operated mice

**Fig. 3:**
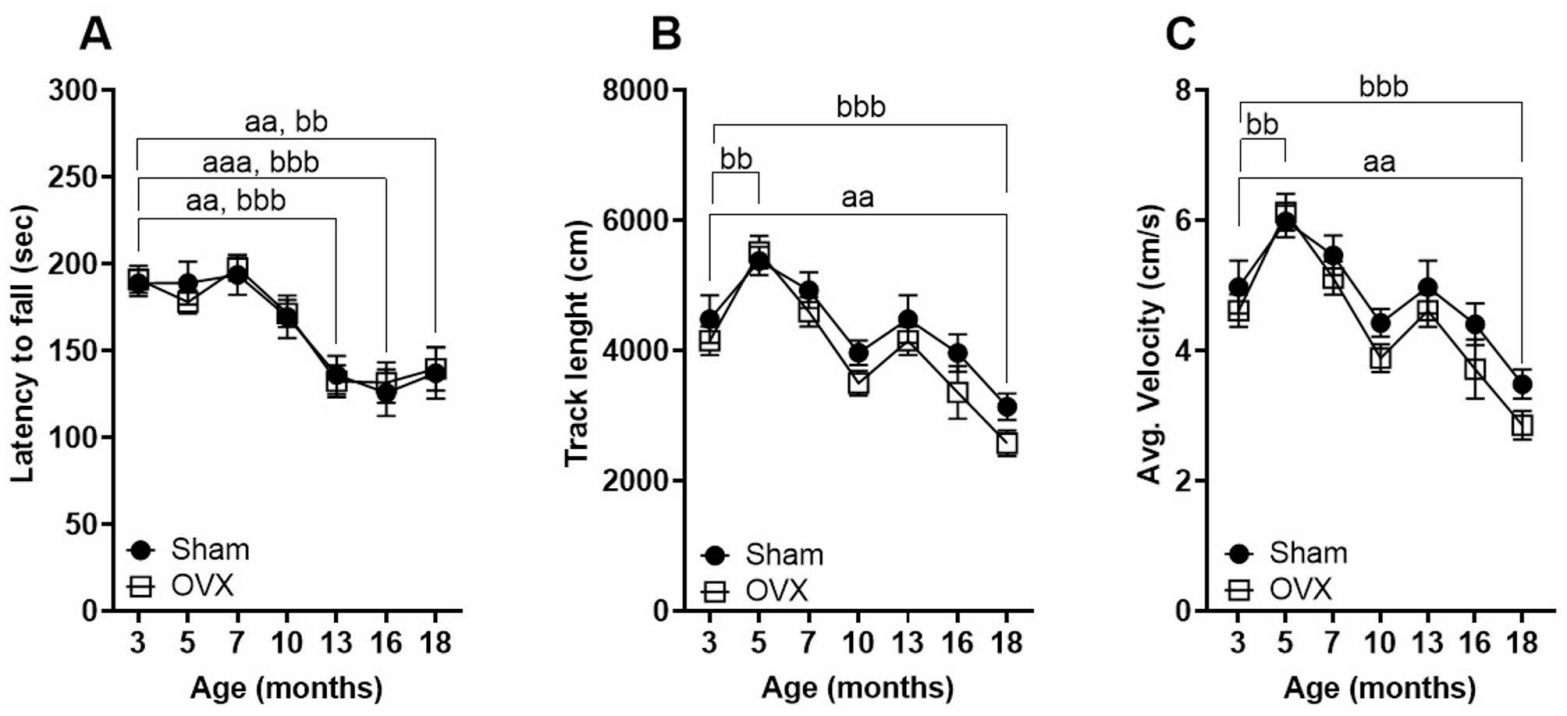
Age-related decline in motor activity is not exacerbated by OVX. Forced (**A**) and spontaneous (**B**–**C**) locomotor activity progressively declined with age in both ovariectomized and sham-operated mice. OVX did not significantly affect motor performance compared to controls at later stages of life. Data are presented as mean ± SEM. Statistical significance was determined by two-way ANOVA followed by Dunnett test. a, indicates statistical difference in the sham group; b, indicates statistical differences in the OVX group. a or b: *p* < 0.05, aa or bb: *p* < 0.01; aaa or bbb < 0.001

Both ovariectomized and sham-operated control mice exhibited a similar progressive decline in forced locomotor activity over time, with a significant reduction observed at 13 months of age (latency to fall, sham = 136 ± 42 s and OVX = 133 ± 36) compared to baseline levels at 3 months (latency to fall, sham = 189 ± 29 s and OVX = 191 ± 30 s). However, performance on the rotarod remained stable from 13 to 18 months of age (Fig. 3A).

Similarly, in the open field test, spontaneous locomotor activity, measured by average velocity and total track length, initially improved at 5 months of age but subsequently declined. By 18 months, both parameters were significantly reduced compared to baseline values recorded at 3 months of age. The ovariectomy did not worsen the spontaneous locomotor activity (Fig. 3B, C).

### OVX transiently increases sensitivity to mechanical stimuli within a defined age window

**Fig. 4:**
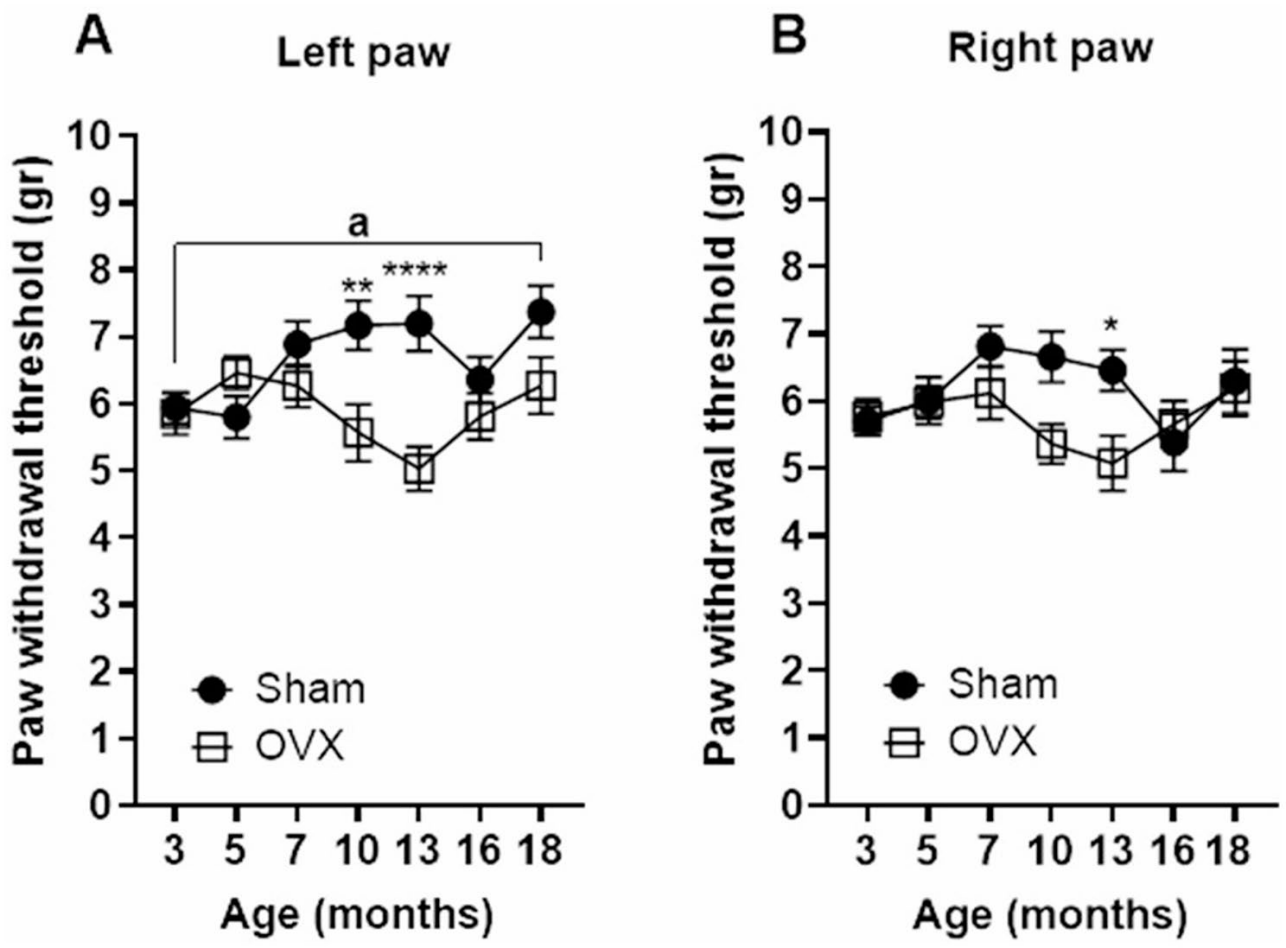
OVX transiently increases mechanical pain sensitivity. Paw withdrawal thresholds in response to mechanical stimuli were assessed in sham-operated and OVX mice from 3 to 18 months of age by using an automated Von Frey test. Significant differences between OVX and sham groups were observed at 10 and 13 months in the left paw and at 13 months in the right paw. Data are presented as mean ± SEM. Statistical analysis was performed using two-way ANOVA with a Dunnett post hoc test. a, indicates statistical difference in the sham group; *, represent statistical differences between Sham and OVX mice; *, *p* < 0.05; **, *p* < 0.01; ****, *p* < 0.001

Sham-operated mice exhibited a gradual decrease in sensitivity to mechanical stimuli over time, with a significantly higher paw withdrawal threshold observed at 18 months compared to baseline (3 months). In contrast, ovariectomized mice showed a transient increase in mechanical sensitivity, with a pronounced decrease in paw withdrawal threshold peaking at 13 months of age. When compared to sham controls, OVX mice displayed significantly lower withdrawal thresholds at 10 and 13 months of age in the left paw, and at 13 months in the right paw (Fig. 4).

### OVX mice show a reduction of the bone mineral density in the trabecular and cortical bone parameters of the tibia

**Fig. 5:**
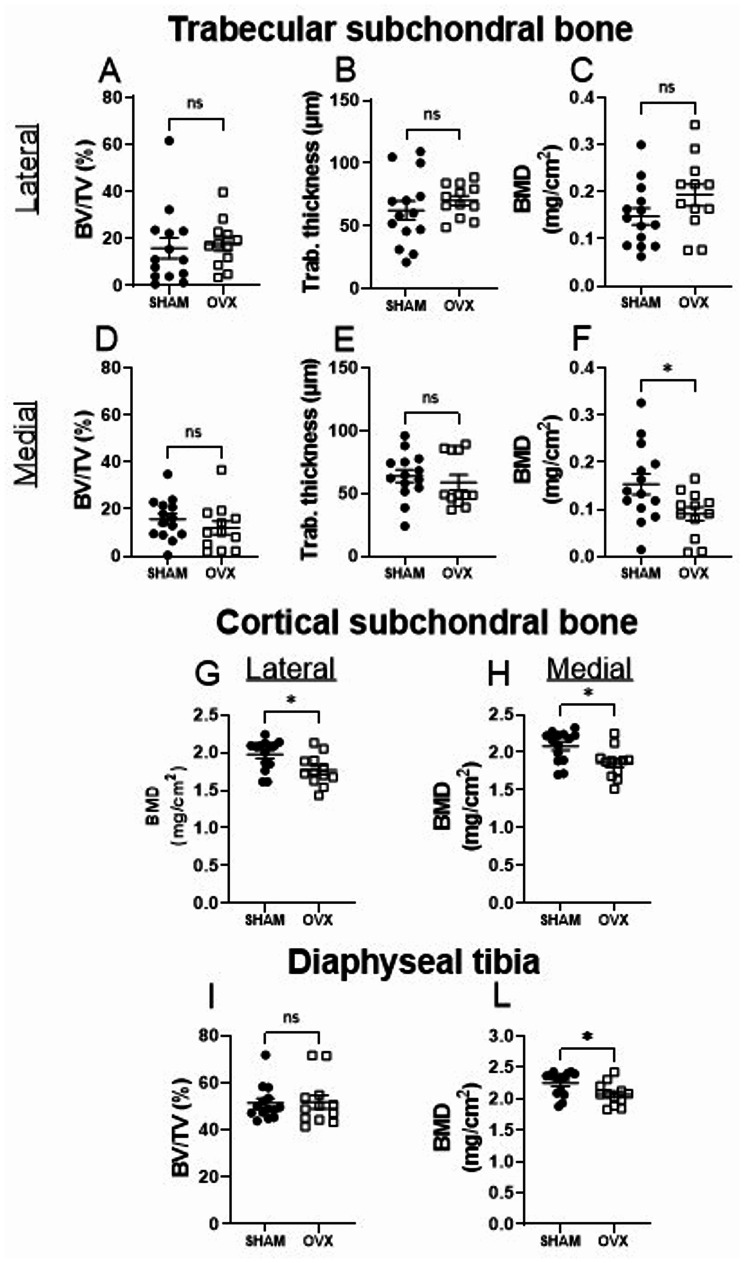
OVX mice show a reduction of the trabecular bone mineral density in the medial subchondral bone, and in the cortical bone of the subchondral and diaphyseal area. Micro-CT analysis of trabecular subchondral bone on the lateral (**A**–**C**) and medial (**D**–**F**) sides, and cortical bone of the subchondral area (**G**, **H**), and diaphyseal tibia (**I**, **L**) in sham-operated and OVX mice. Data are presented as mean ± SEM. Statistical significance was determined using an unpaired Student’s t-test. *, represent statistical differences between Sham and OVX mice; *, *p* < 0.05

Tibial subchondral bone of sham-operated and OVX mice were analyzed by µCT. No differences are measured in the two experimental groups for the trabecular bone volume (BV/TV) and trabecular thickness (Fig. 5), whereas the bone mineral density (BMD) in the medial side declines in the OVX mice compared to the sham-operated (Fig. 5A-F). Similarly, in OVX mice the BMD of the cortical subchondral region and of the diaphyseal area was significantly reduced compared to the sham operated mice (Fig. 5G-L).

### Trabecular bone is reduced in the vertebrae of OVX mice compared to sham-operated controls

**Fig. 6:**
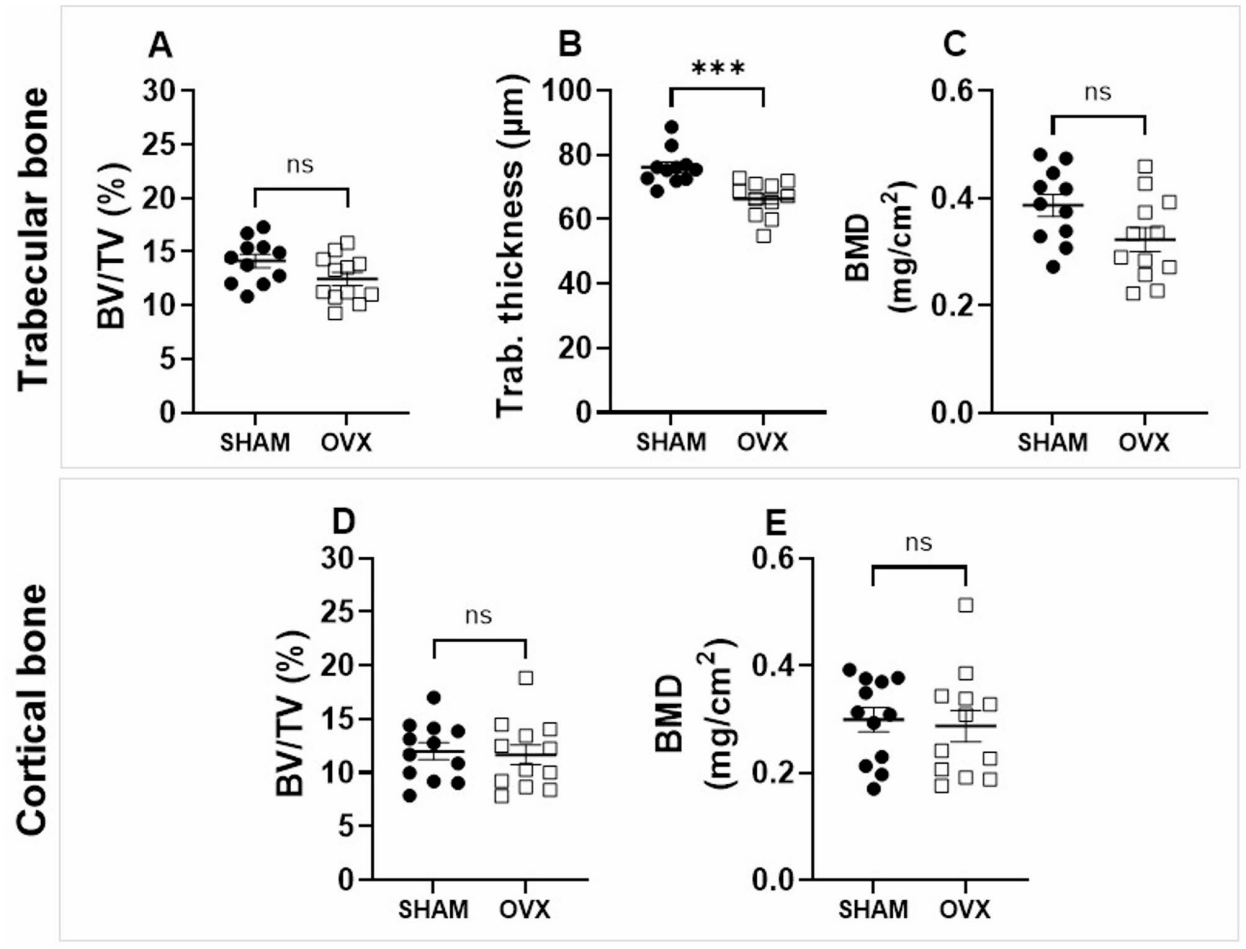
Vertebral trabecular bone is reduced in OVX mice. Micro-CT analysis of trabecular (**A**–**C**) and cortical (**D**, **E**) bone in the vertebrae of sham-operated and OVX mice. Data are presented as mean ± SEM. Statistical significance was determined using an unpaired Student’s *t*-test. *, represent statistical differences between Sham and OVX mice; ***, *p* < 0.001

Micro-CT analysis was performed on the L4 vertebrae to assess the effects of ovariectomy on vertebral bone structure. The results showed a decline in trabecular bone volume, trabecular thickness, and bone mineral density in OVX mice relative to the sham-operated group (Fig. 6A–C), indicating substantial trabecular bone loss. In contrast, no significant differences were detected between the two groups in cortical bone parameters (Fig. 6D, E).

### Articular cartilage exhibited a similar proteoglycan loss in both experimental groups

**Fig. 7:**
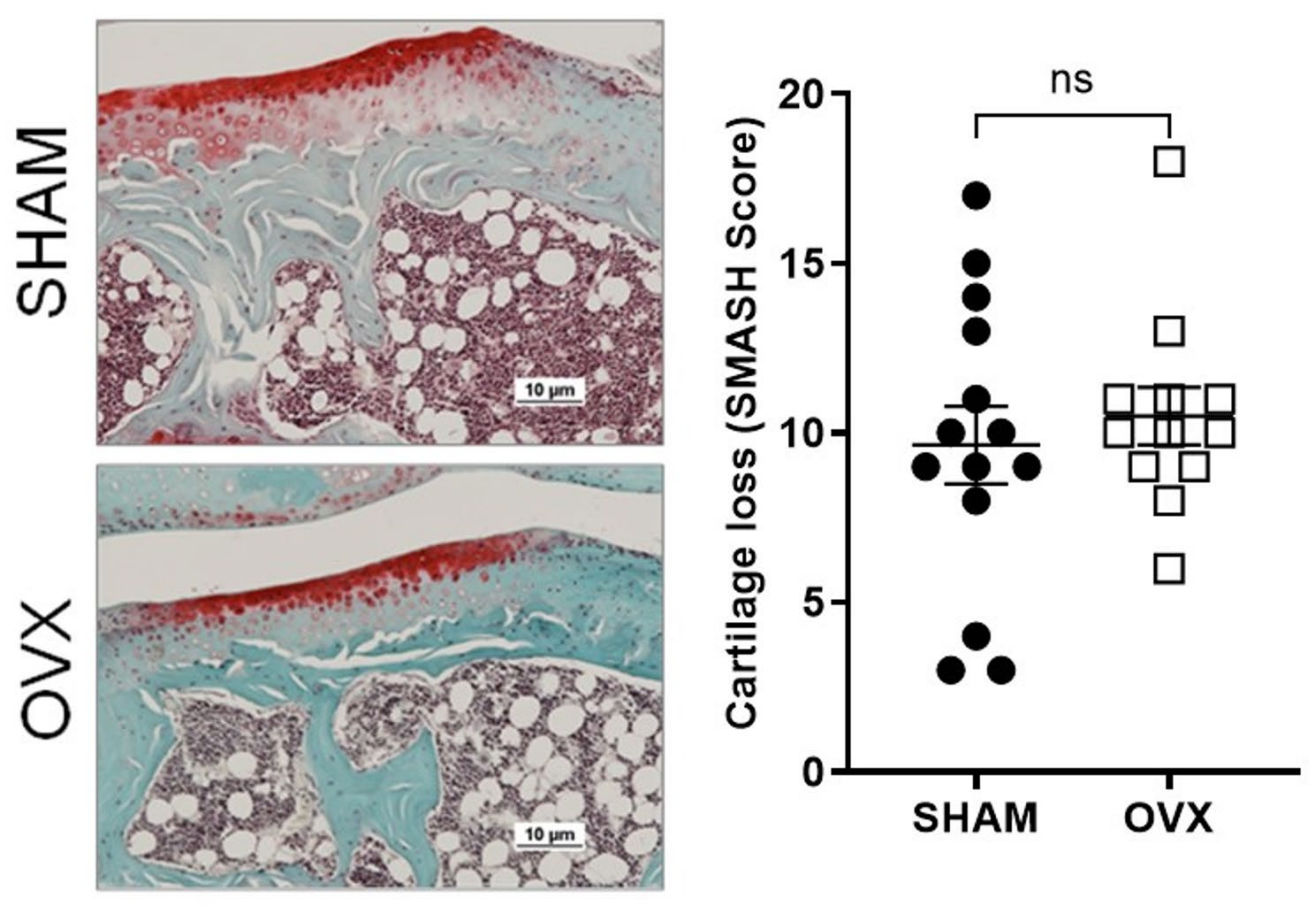
Age-related proteoglycan loss was similar in OVX and sham-operated mice. Representative Safranin-O staining and semiquantitative assessment of proteoglycan depletion (SMASH score) in the articular cartilage of the knee joint from OVX and sham-operated mice. Data are presented as mean ± SEM. Statistical significance was assessed using an unpaired Student’s *t*-test

Knee articular cartilage was analyzed using Safranin-O staining. Both experimental groups exhibited proteoglycan loss. Semiquantitative analysis of the cartilage matrix (SMASH score) showed a similar score between the experimental groups (Fig. 7).

## Discussion

This study aimed to investigate the long-term effects of ovariectomy (OVX) in adult female mice on joint health, motor function, and pain sensitivity, all key features of knee osteoarthritis, which has a higher incidence in postmenopausal women [15]. In humans, menopause is a gradual transition marked by fluctuating gonadal hormone levels that eventually stabilize at low concentrations during the perimenopausal period, which typically lasts 4–7 years [16]. Evidence indicates that hormonal fluctuations, rather than the absolute decline, underlie the increased predisposition to several age-related diseases [17, 18]. Although ovariectomy (OVX) is a widely used and well-established model of the postmenopausal state, it does not replicate the perimenopausal condition, as it induces an abrupt decline in gonadal hormones [19].

To better model the postmenopausal condition and incorporate aging as a contributing factor, we performed OVX on 7-month-old mice.

In our study, OVX does not worsen the age-related decline of the motor function. Despite prior evidence linking estrogen depletion to impaired muscle strength, skeletal disorders, and neurodegeneration [20–22], our results show that OVX in adult mice did not exacerbate the age-associated decline in either forced or spontaneous locomotor activity. Similar to our study, in a previous study on 5-month-old mice ovariectomized at 8 weeks of age, no differences were measured between OVX and control mice in the forced locomotor activity. However, the same study showed an increased travel distance in the open field test in the OVX mice compared to the sham-operated, attributed to anxiety behavior [23]. In our previous study on 4-month-old animals OVX at 8 weeks of age, no differences were detected in the travel distance in the open field test, and a significant reduction in the forced locomotor activity was measured [19]. Our current results suggest that by 7 months of age, mice have already begun to experience age-related motor changes, possibly masking additional effects of OVX. Indeed, spontaneous and forced locomotor activity in C57BL/J mice progressively declines starting at 3 months of age [24]. It’s also plausible that adaptive mechanisms following OVX mitigate long-term impairments in motor function.

We instead observed a transient increase in mechanical pain sensitivity in OVX mice, particularly between 10 and 13 months of age. These results support previous evidence that estrogen modulates pain perception [25] and suggest that timing post-OVX is critical in assessing pain outcomes.

Contrary to what is typically seen in young ovariectomized mice, we found no significant difference in body weight or perigonadal fat accumulation. Young ovariectomized mice show an increase in visceral and subcutaneous fat, not associated with a reduction in physical activity or increased food intake [26]. This discrepancy with our findings may be due to age-related metabolic changes [27] that blunt the lipogenic effects of estrogen loss. Only uterine atrophy confirmed sustained estrogen deficiency similar to that observed in the ovariectomized young animal [17]. The thymus, on the other hand, showed a reduced mass, in contrast to what has been reported in young animals, where its weight increases following ovary removal [28]. Consistent with studies in young mice, OVX induced significant reductions in bone mineral density (BMD) in the medial tibial subchondral region, cortical bone, and vertebral trabecular bone [19]. However, unlike in younger models, there was no effect on trabecular bone volume or thickness in the knee joint. These observations are consistent with the notion that trabecular bone loss in response to OVX is more pronounced when initiated early in life, as bone remodeling activity diminishes with age [29]. The reduction in BMD in ovariectomized mice on the medial side may reflect the greatest biomechanical adaptation in OVX mice to the morphological changes in the tibia occurring during aging [30].

This biomechanical adaptation in ovariectomized mice did not impact cartilage integrity. Histological analysis of articular cartilage did not reveal increased proteoglycan loss in ovariectomized mice compared to controls. Both experimental groups exhibited signs of age-related cartilage degeneration as previously shown [31], suggesting that gonadal hormone deficiency in aging mice does not affect the cartilage physiology.

## Conclusions

Our findings provide new insights into how aging and loss of ovarian hormones interact in modulating musculoskeletal function and integrity.

This study demonstrates that ovariectomy in adult female mice induces selective changes in bone and pain sensitivity without markedly altering age-related motor decline, fat deposition, and cartilage health. These results reinforce the need to incorporate aging as a variable in preclinical models of postmenopausal diseases and suggest that OVX in older animals offers a more clinically relevant paradigm for studying idiopathic osteoarthritis.

## Data Availability

The datasets analyzed during the current study are available from the corresponding author upon reasonable request.
